# The effect of sub-facet scale surface structure on wall brightness temperatures at multiple scales

**DOI:** 10.1007/s00704-020-03094-7

**Published:** 2020-02-05

**Authors:** Rainer V. J. Hilland, James A. Voogt

**Affiliations:** 1 Department of Environmental Meteorology, Albert-Ludwigs Universität Freiburg, Werthmannstraße 10, D-79085 Freiburg, Germany; 2 Department of Geography, Western University, 1151 Richmond St, London, ON N6A 3K7 Canada

## Abstract

Wall surface temperatures are important components of urban climates but are under-sampled by satellite and airborne remote sensing and at the microscale are under-sampled in observational studies. In urban canopy models, they are represented with simplistic geometries. This study examines the effect of microscale (sub-facet) surface structure geometries on wall surface brightness temperature distributions at micro- to neighbourhood scales using mobile sampling traverses of two suburban neighbourhoods with different sub-facet geometries. Visible and thermal imagery were recorded simultaneously and combined and classified to create a database of temperatures with associated geographic and thermal attributes. This study investigates (1) if sub-facet scale geometries affect temperature distributions, (2) if these cause canyon scale biases, and (3) if there are therefore inter-neighbourhood biases. It is shown that sub-facet geometries modify wall surface temperatures predominantly by cooling due to self-shading. Surface-sun geometry thus leads to intra- and inter-neighbourhood temperature differences of several degrees Celsius. The observed effects have important implications for modelling of urban surface temperatures, where simplified geometries may overestimate wall surface temperatures.

## Introduction

### Context

The materials and geometries of urban environments create a unique urban climate typified by higher surface and air temperatures relative to their non-urban surroundings (Oke et al. 2017). Urban surface temperatures play important roles in the experienced urban climate, affecting human health and comfort, building energy use and internal temperature, as well as urban wind flows.

Of the individual roof, street, and wall facets that create the urban form, walls can form a signification portion, ranging from 12 to over 50% of the complete surface area (Voogt and Oke 1997; Grimmond and Oke 1999). Despite their large surface area, many methods of thermal remote sensing have a tendency to under-sample wall surfaces. Remote observation methods that view the surface from a (near) nadir angle will preferentially view horizontal surfaces such as roofs and roads. Deliberate observations are necessary to ensure that vertical surfaces are not overlooked.

Further, models often simplify the representation of urban surface structure so that facets such as walls and streets may be represented as homogenous plane surfaces, omitting small-scale structure features on facets such as overhangs, awnings, doors, and windows. These features exist at the sub-facet scale and add complexity to the urban surface structure. In the language of three-dimensional building models like CityGML, these features correspond roughly to Level of Detail 3 (Gröger and Plümer 2012). Here we describe the features as sub-facet scale surface structure or more simply as microscale geometries. Surface structure impacts the surface energy balance and hence the surface temperature of the facet, but it is not known if the omission of these structures leads to model biases in estimates of surface temperature.

### Observational studies

As a component of many urban microclimate processes, wall temperatures are regularly measured or factored in to observational campaigns or microscale climate simulations (e.g. Best and Grimmond 2015; Offerle et al. 2006; Pigeon et al. 2008; Rotach et al. 2005). Despite their ubiquity as a component of research studies, there has been little work that observes wall temperatures and their variability at a range of spatial scales, or the microscale geometries of walls themselves.

Offerle et al. (2006) measured wall temperatures of a cross-section of a street canyon using a series of contact thermocouples. Although mean facet temperatures progress along the diurnal trend expected due to simple sun-surface geometry, there is evidence that the mean value is inadequately representing sub-facet variability (Offerle et al. 2006, p. 288) and this may be due to structural variability on the facet that is not represented by the smooth surface of the conceptual urban canyon. Sampling temperatures in a canyon cross-section can provide high temporal resolution but does not resolve variability along the canyon axis. Niachou et al. (2008) used a similar cross-section sampling approach with a handheld infrared thermometer. Their canyon incorporates a large amount of sub-facet structural variation making it likely that shading from sub-facet scale structures complicates temperature measurement. Pigeon et al. (2008) sampled facet temperatures in multiple canyons from fixed locations with infrared radiometers to yield a combined wall temperature for comparison to Town Energy Balance model output. Site selection considered the street orientations necessary to provide an appropriate spatial average and selected individual monitoring selections that best matched model assumptions, thus avoiding buildings with more complicated facet geometry.

Another approach to examining walls at the sub-facet scale is time-sequential thermography (TST). TST involves the use of a fixed thermal imager, a high sampling rate (once per second to once per 5 min) and a long sampling period. Hoyano et al. (1999) observed two buildings over the course of a year to capture seasonal variation and characterize the sensible heat fluxes. Canyon radiative temperatures were captured at 1- and 5-min intervals during the BUBBLE (Basel UrBan Boundary Layer Experiment) campaign (Rotach et al. 2005) from a fixed imager, though the views of canyon walls were relatively oblique. During this campaign, Aldred (2003, unpublished thesis) examined sub-facet variability of wall surface temperatures using snapshots of canyon walls. Christen et al. (2012) similarly used TST at a high (1 Hz) temporal resolution to consider microscale temperature variability of, among others, wall facets in Berlin. TST provides the option of extremely high spatio-temporal resolution of urban facet temperatures but is conducted from a fixed platform. This limits the ability of the approach to categorize wall temperature variability at the neighbourhood scale.

An improvement to the spatial limitation of a fixed thermal imager is panoramic time-sequential thermography (PTST) described in Adderley et al. (2015). Here an articulating thermal imager is able to pivot and rotate to capture thermal images over a full 360° range of azimuth and a large range of off-nadir angles in a short period of time. This sampling technique allows for the densest spatio-temporal scales of observations to date but still preferentially views horizontal surfaces, and its fixed location and limited height restrict the spatial scale over which surfaces can be viewed.

To sample wall temperatures at larger spatial scales requires mobile sampling approaches. Voogt and Oke (1998) explicitly examined wall temperatures within and across different neighbourhoods. The study traversed a vehicle mounted with infrared radiometers (IRRs) aimed at the canyon walls through various neighbourhoods. Observations yielded temperature distributions of vertical facets but the non-scanning 15° field of view (FOV) instruments record one temperature that is an aggregate of all surfaces within the FOV and is thus unable to resolve sub-facet scale temperature variability and involves deriving facet temperatures from distributions that likely include samples contaminated by non-wall surfaces.

### Modelled wall facet temperatures

The rise of computer power has driven computer simulations to the forefront of much research in the urban climate literature, and surface temperatures are no exception. There are many urban canopy models currently in use for a variety of applications that derive wall temperatures (Best and Grimmond 2015). These models operate at a variety of scales but tend to produce mean facet temperatures as outputs.

Temperatures of Urban Facets in 3D (TUF-3D) is a model that resolves the surface energy balance at a sub-facet scale (Krayenhoff and Voogt 2007) and has been employed to study thermal phenomena related to urban geometry (Krayenhoff and Voogt 2016). Although the model has shown good agreement when compared against observational data, it has also overestimated wall temperatures (Krayenhoff and Voogt 2007, p. 454). It is hypothesised that this disagreement may be due to sub-facet shading from the surface geometry: in the TUF-3D model, although temperatures may be resolved at the sub-facet scale, the facets themselves are all considered to be smooth surfaces with no geometric variation, an assumption that is common to almost all urban canopy models (Best and Grimmond 2015). Lindberg et al. (2015) found that sunlit fractions on walls were overestimated when using simple infinitely long canyons but were improved when using more realistic building height and shape information. Yaghoobian and Kleissl (2012) modified TUF-3D to incorporate sub-facet scale variability by including windows for the purpose of building energy simulation. This method modifies the thermal and radiative properties of sub-facet patches but does not address geometric complexity of the facet.

To date studies of urban wall temperatures have generally failed to consider the temperature variability that occurs at a sub-facet scale. The small body of work that has considered sub-facet scale temperatures has not addressed the effects of sub-facet geometry on wall temperature patterns and the extent to which such effects might bias the representation of wall temperatures in numerical models of urban surface temperature where facets are represented as smooth surfaces.

Here, we take the first step in filling this gap by examining sub-facet scale temperatures at high spatial resolution over a diurnal cycle. A mobile traverse approach is adopted to sample wall temperatures at high spatial resolution over spatial scales that incorporate the important building scale variability within two select neighbourhoods that differ in the complexity of their sub-facet wall geometries. Using a series of traverses over a clear day, we generate a unique dataset that allows us to answer the following research questions:

1. Does sub-facet scale surface structure affect wall facet temperature distributions?
2. Are there wall temperature biases at the canyon scale due to sub-facet scale surface structures?
3. Do neighbourhoods with different types and sizes of sub-facet scale surface structures exhibit different wall temperature distributions due to these geometries?

Section 2 of this paper describes the study sites and methods. Section 3 discusses the analysis and results at facet, canyon, and neighbourhood scales. The scale terminology used here is adopted from that used in Oke et al. (2017). Section 4 situates the results within the literature and discusses implications.

## Methods

### Site selection and description

Two residential neighbourhoods with differing facet scale structures were selected in London, Ontario, Canada. The first site (S1) is characterized by houses with large covered porches, which create large areas of wall shading at most times of day. In contrast, the houses of the second site (S2) have few sub-facet scale structures to cause self-shading. Sites were selected to minimize travel time between the sites and the number of trees between the street and the houses, which would cause shadows and therefore facet scale temperature variability as well as contain houses with similar orientations. The street grid in both neighbourhoods is rotated approximately 20° counter-clockwise from North, but for ease of discussion, this paper will refer to facets as facing north, east, south, or west. The neighbourhoods were sampled sequentially. The driving distance between the two sites is approximately 4.5 km.

**Fig. 1:**
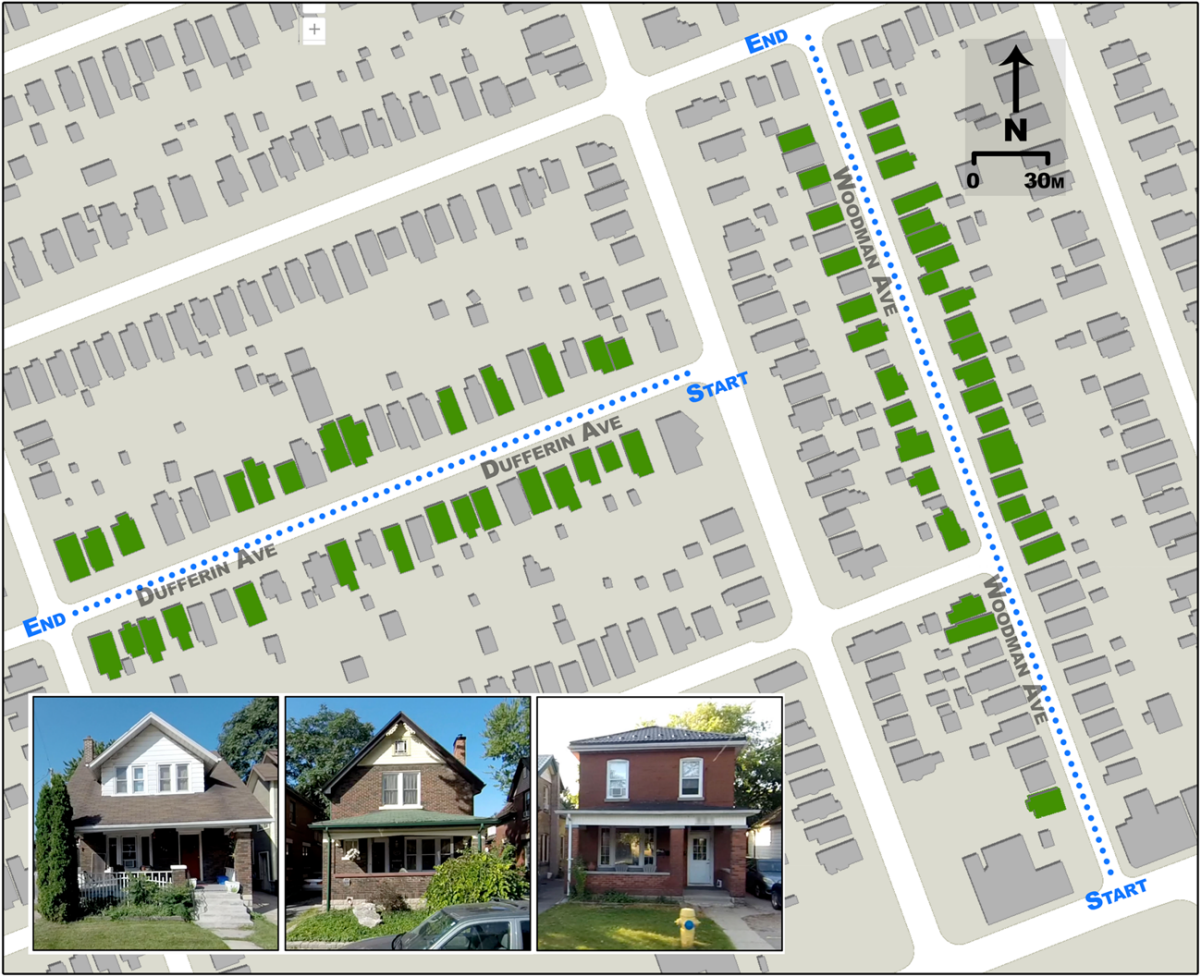
Map of S1. Dotted line shows the traverse paths and were driven both directions. Buildings coloured green are included in the final dataset (Section 2.3.1). Inset are sample images of representative houses

S1 (Fig. 1) is located in the Old East Village neighbourhood (42°59′40.84” N, 81°13′12.06” W). The site comprises the entirety of the N-S street canyon of Woodman Ave, and a one block subset of the E-W street canyon of Dufferin Ave. In total this site contains 100 houses: 25 east-facing, 30 west-facing, 22 north-facing, and 23 south-facing. The houses are relatively older, with most built between 1920 and 1940 as determined from aerial photography archives, though approximately 25% of the houses predate 1920. Of the 100 houses, 90 have porches or overhangs that cover some or all of the first storey. Houses include a mixture of building materials and colours but are predominantly brick or vinyl-sided.

**Fig. 2:**
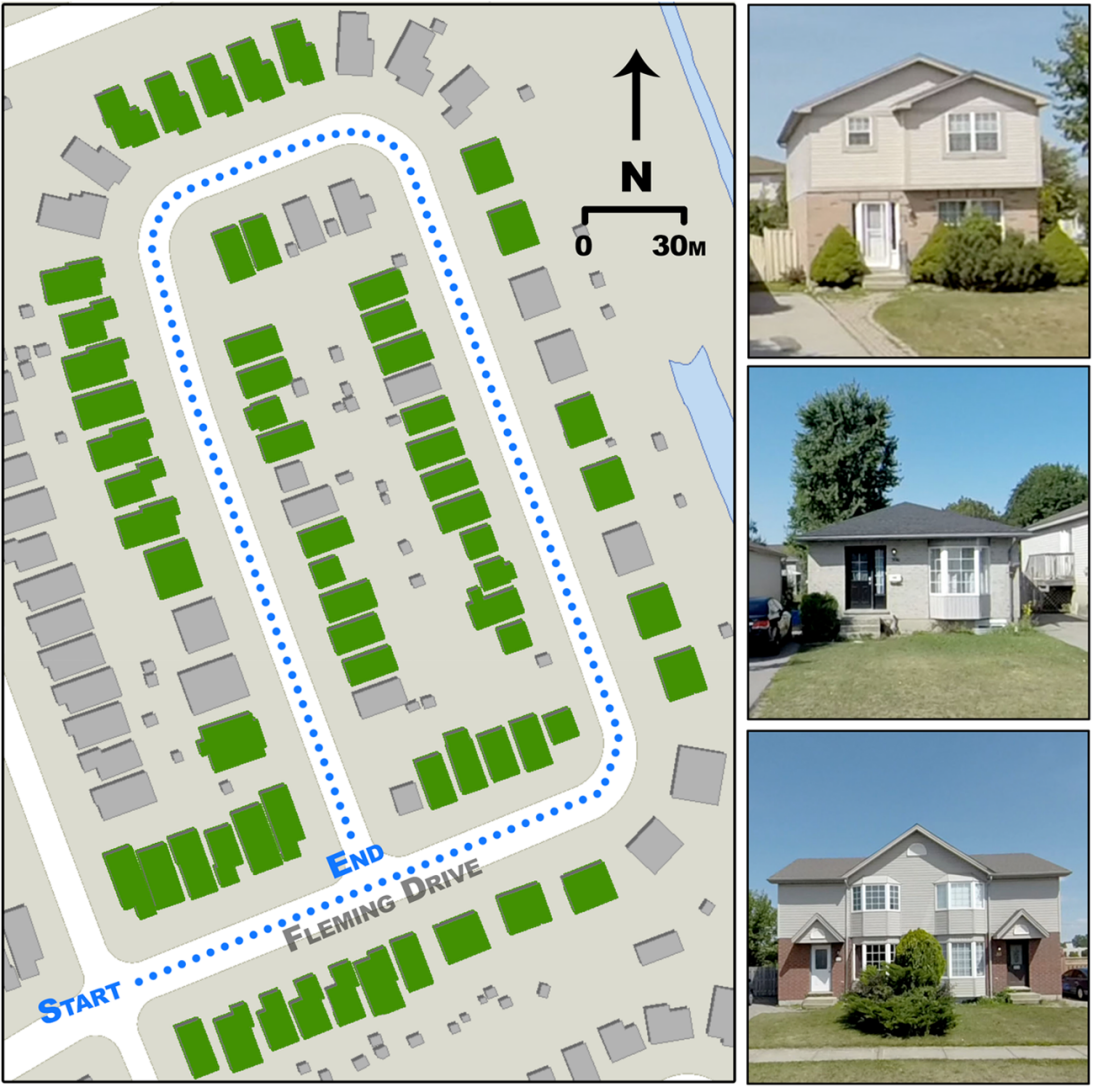
Map of S2. Dotted line shows the traverse path and was driven both directions. Buildings coloured green are included in the final dataset (Section 2.3.1). Accompanying photos are sample images of representative houses

S2 (Fig. 2) is located in the Huron Heights neighbourhood (43°01′02.85” N, 81°11′44.31” W). The site comprises the entirety of Fleming Drive, a street which consists of a loop and encompasses all four cardinal directions. The site consists of 82 houses: 24 east-facing, 23 west-facing, 15 north-facing, and 20 south-facing. The houses were constructed in 1996–1998, and only four have porches that cover the first storey. There is a mixture of one- and two-storey buildings, as well as a mixture of brick and vinyl-siding.

S1 has an average canyon height to width ratio (H:W) of roughly 0.25; the H:W for S2 varies between 0.2 and 0.1 depending on building height. Differences in canyon H:W affect both access to direct and diffuse short-wave radiation, and the rate at which a neighbourhood will cool by modifying the sky view factor (SVF) (Oke 1982), thereby influencing wall temperatures to some degree. Simulations using TUF-3D suggest that the H:W difference between S1 and S2 accounts for < 1.5 °C difference in mean facet temperature at any given time of day (see Section A.2).

### Field methods

#### Fixed site

To obtain boundary conditions and forcing data for modelling, a fixed monitoring site was erected on the roof of Talbot College on the Western University campus (43°00′27.38” N, 81°16′14.16” W). Above-canopy incident short-wave and long-wave radiation (K ↓, L ↓) and air temperature at reference height z (T_air_) were collected. The instruments and variables obtained are summarized in Table 1. All instruments were sampled once per second and 5-min averages were recorded.

**Table 1:** Instrumentation

Platform	Instrument	Manufacturer	Variable(s) (units)	Nominal accuracy	Nominal response time
Fixed	HC2-S3 Probe	Campbell Scientific	*T*_*air*_ (°C) RH (*%*)	± 0.1 °C ± 0.8%	< 22 s
	CMA 6 Albedometer	Kipp & Zonen	*K*↓ (W m^−2^)	± 0.1%	< 6 s (63*%)*
	Precision Infrared Radiometer	Eppley	*L*↓ (W m^−2^)	± 0.5%	5 s (95%)
Mobile	T650 Thermal Imager	FLIR	*T*_*wall*_ (°C)	See Table 2	
	HC2-S3 Probe	Campbell Scientific	*T*_air_ (°C) RH (*%*)	± 0.1 °C	< 22 s
	16X-HVS GPS	Garmin	Location	± 0.8*%* <15 m	1 s
	Hero 3+ (TLC)	GoPro	RGB photos		
	ST-200 Fine-wire thermistor	Apogee	*T*_*air*_(°C)	±0.2 °C	1 s

Measurements of wind speed and direction (*u*, φ) were obtained from the London International Airport and transformed via Eq. 1 as per Wieringa (1986).

1 $$ {\overline{u}}_{zA}=\left[\frac{\ln \left(\raisebox{1ex}{${z}_r$}\!\left/ \!\raisebox{-1ex}{${z}_{0B}$}\right.\right)\ln \left(\raisebox{1ex}{${z}_A$}\!\left/ \!\raisebox{-1ex}{${z}_{0A}$}\right.\right)}{\ln \left(\raisebox{1ex}{${z}_B$}\!\left/ \!\raisebox{-1ex}{${z}_{0B}$}\right.\right)\ln \left(\raisebox{1ex}{${z}_r$}\!\left/ \!\raisebox{-1ex}{${z}_{0A}$}\right.\right)}\right] $$

where $\overline{u}$ is mean wind speed, z_r_ is the blending height (approximated as two times the mean building height), z_0_ is the roughness length, and the subscripts A and B refer respectively to the site of interest and the site of observation. Roughness length at the airport was 0.005 m, a tabled value for grass, and one tenth of the average height of roughness elements for sites A and B (0.75 m) (Oke 1987). These transformed values were then linearly interpolated to a 5-min interval for use in the forcing file for TUF-3D modelling.

#### Instrumented vehicle traverses

Observations at the study sites were conducted using a pickup truck as an instrument platform (Table 1). Such a mobile platform allows the full extent of the sites to be sampled in the smallest possible time, i.e. minimizing the elapsed time from the start to the end of a sampling period. The primary instrument used in this research is a FLIR T650 thermal imager (Table 2). Imagery is also collected in visible wavelengths using a GoPro digital action camera configured as a time-lapse camera (TLC). With the exception of the thermal imager, all instruments were sampled at 1 Hz. The thermal imager was positioned inside the vehicle cabin and recorded out of the rear left-facing window at 5 Hz. The thermal imager allows sub-facet scale temperature variations to be resolved and clearly delineates surfaces of interest from those that are extraneous (e.g. background sky). This allows for removal of unwanted temperature measurements. It also enables the classification of pixels (temperatures) according to any desired set of variables, i.e. subsets of an individual thermal image can be associated to a set of classification criteria. By combining the thermal imagery with the RGB imagery from the TLC, we can more precisely determine characteristics of the surface under consideration. The TLC provides an additional spectral channel so that nominal variables such as surface material, colour, shading, etc. that cannot be accurately ascertained from the thermal image alone can be classified by using the RGB image of the house as a reference.

**Table 2:** Specifications of the FLIR T650 thermal imager (FLIR 2016a)

Parameter	Value
Temperature range	− 40 to 150 °C
NETD	< 20 mK
Accuracy	±1 °C
Spectral range	7.5–13.0 μm
Time constant	< 8 ms
Resolution	640 × 480px
Detector	Uncooled microbolometer
Focal length	13.1 mm
FOV	45^°^ × 34^°^

The radiance received by the thermal imager’s sensor array is converted to a radiometric surface temperature using FLIR (2016b); however, for this paper, raw radiance is converted directly to temperature and referred to as directional brightness temperature, as per Norman et al. (1995). Minkina and Dudzik (2009) show that at atmospheric temperatures, humidities, and path lengths similar to those measured during this project (10–20 m), the atmospheric influence on measured temperature is <0.01 K, below the accuracy of the instrument. Additionally, this paper concerns itself with temperature distributions and differences, which are not significantly affected by differences in ε when surface materials share similar emissivities. Tabled values of emissivity for the two most common materials of brick and vinyl siding (> 70% of surfaces) fall within the same range (Porter 2009, upublished MSc thesis). Glass surfaces are excluded from analysis as the wide range in emissivities makes radiative temperature measurements impractical.

Each traverse encompassed both sites. Eight traverses were conducted (6 daytime, 2 night-time) over the course of a 24 h period to consider the diurnal progression of surface temperature and shading patterns. Table 3 shows the traverse numbers and timing of each as recorded by the GPS. The daytime traverses were selected to produce a variety of shading patterns on the houses, and the night-time traverses so that differences in cooling processes between neighbourhoods could be considered.

**Table 3:** Traverse numbers (1–8) and times (HH:MM:SS) for each site (1, 2) and associated solar elevation (β) and solar azimuth (Ω) angles at the start and end of each traverse

Traverse	Site	Start time (EDT)	End time (EDT)	*β*_*start*_ (deg)	*β*_*end*_ *(deg)*	Ω_start_ (deg)	Ω_end_ (deg)
1	1	08:12:00	08:20:18	15.2	19.1	90.1	93.8
	2	08:29:19	08:33:30				
2	1	10:06:58	10:13:01	35.7	38.9	111.8	116.2
	2	10:21:37	10:26:05				
3	1	11:43:38	11:49:15	50.2	52.2	137.9	144
	2	11:57:33	12:01:11				
4	1	14:12:45	14:17:48	55.6	54.4	200.4	207.3
	2	14:25:21	14:29:37				
5	1	16:35:18	16:40:46	37.5	34.4	245.5	249.7
	2	16:49:37	16:53:54				
6	1	18:41:21	18:58:34	15.1	11.9	269.7	272.7
	2	18:54:04	18:58:34				
7	1	23:35:05	23:43:00	Night-time			
	2	23:51:14	23:55:35				
8	1	05:05:30	05:11:06	Night-time			
	2	05:19:14	05:23:48				

Data from the traverses will be discussed as single points in time in this paper, i.e. temperatures from traverse one are considered to have been taken at the same time even though there are roughly 20 min between the sampling of the first and last house. Simulations with TUF-3D suggest that the mean change in temperature of a facet over a 15-min period is 0.3 K and the maximum 1.5 K. This mean temperature change falls within the accuracy of the instrument, though in practice at sub-facet scales, the wall will heat differentially with material. Given the myriad facet properties that control surface temperature, no corrections for temperature change during a traverse are incorporated in the analysis.

#### Kinematic LiDAR measurements

**Fig. 3:**
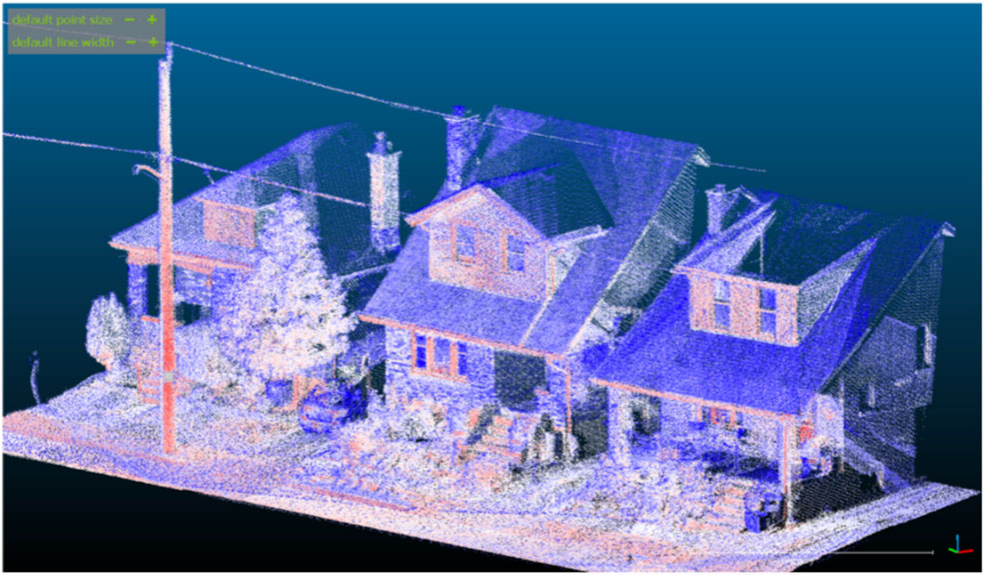
Screenshot illustrating the level of detail provided by the KLS scans. Colour indicates return intensity, with red higher intensity and blue lower intensity

The surface structure of the neighbourhoods was obtained through a Light Detection and Ranging (LiDAR) system. The kinematic LiDAR system (KLS) consists of a backpack-mounted LiDAR scanner, DGPS system, and an inertial measurement unit. The system enables the creation of very dense (> 5000 points m^−2^) point clouds at cm-scale accuracy and samples while the operator walks through the observation site (Galofre et al. 2018; Kukko et al. 2012). The significant advantage of the KLS in comparison to airborne LiDAR systems is its ability to capture the complex 3D geometry of a residential neighbourhood, including vertical surfaces that are hidden from above by, e.g. porches. An example is shown in Fig. 3. Scans were performed on October 5, 2017.

### Data processing

#### Preprocessing

The image series recorded by the thermal imager were examined frame-by-frame, and the clearest, most comprehensive frames of each house were exported as individual CSV files of brightness temperature. Each image was corrected for geometric distortion caused by the lens and imported to ArcGIS. Geometric distortions were corrected using the Camera Calibration Application in the Image Processing and Computer Vision Toolbox of MATLAB (MATLAB 2016) based on a set of 34 thermal images of a custom calibration checkerboard constructed of alternating low and high emissivity surfaces (Hilland 2018). The mean reprojection error was 0.1465 pixels, which satisfies the rule of thumb by Scaramuzza et al. (2006) that errors be less than 1 pixel. A similar process was performed for the TLC imagery: individual frames were selected and geometrically corrected for each house.

Of the 182 houses sampled, 57 were removed for various reasons: 17 houses could not be measured due to an incomplete LiDAR dataset on Woodman Ave caused by equipment malfunction, significant facet obstruction due to vegetation eliminated 33 houses, and 7 houses were excluded from S2 that did not share the same orientations as the other houses. Table 4 shows the result of the excluded houses on the distribution of facets. The remaining 125 houses were processed for inclusion in the final dataset. Five other individual images are missing due to periodic automatic calibration of the thermal imager against its own internal temperature during which the shutter is closed. In those cases, the individual images are excluded but the house remains in the dataset.

**Table 4:** Excluded houses by site number and orientation

Site	Orientation	Starting houses	Remove due to:			Remaining houses
			Obstruction	LiDAR	Orientation	
1	East	25	4	7	–	14
	West	30	–	10	–	20
	North	22	17	–	–	15
	South	23	10	–	–	13
2	East	24	3	–	1	20
	West	23	6	–	2	15
	North	15	2	–	1	12
	South	20	1	–	3	16
Total		182	33	17	7	125

#### Image co-registration

Both visible and thermal images were co-registered to a common local coordinate system (LCS) for each house. Due to the precision provided by the kinematic LiDAR scans, a centimetre-scale LCS was used. It provided ordered (*x*, *z*) coordinate pairs with constant (*y*) distance from the imager. Measurements of the dimensions of each street-facing facet in cm were made from the LiDAR point clouds, and a series of pseudo-ground control points (GCPs) were collected. Due to the three-dimensionality of the structures under consideration, GCPs were only selected from a single vertical plane of the facet, and the rectification can only be considered accurate on this plane. Surfaces that extend to the foreground from the main facet plane (smaller *y*), such as porch pillars, as well as surfaces that extend to the background (greater *y*), such as rooftops, are often poorly placed. Their location in the image depends highly on the angle between the imager and the facet.

The visible imagery from the TLC was co-registered first using the GCPs, and the thermal imagery subsequently referenced to the visible imagery. The reasons for this are twofold: first, most facets from the first study site were too large to fit in a single thermal image and therefore two or more images needed to be mosaicked together. Co-registering each individual image would necessitate the collection of at least twice as many GCPs per facet. Secondly, a primary purpose in combining the visible and thermal imagery was to use the visible imagery as an independent assessment of surface characteristics and shadow locations on the thermal imagery, making it more important that the images align with each other rather than fit perfectly to the LCS. With few exceptions, the images were transformed using an affine transformation. The best fit for each image was determined subjectively on a case-by-case basis that attempted to (a) create the best alignment in the primary vertical plane so that shadow patterns could be most accurately determined, (b) create the least visual distortion of the imagery, and (c) minimize the re-projection error.

Facets for which multiple thermal images were needed had their component images mosaicked to form a single image. To avoid distorting actual temperature values, the images were not combined with a ‘blend’ or ‘mean’ function; this results in a seam through each composite image. The seam could be navigated during classification of the image, without affecting the temperature measurements. During mosaicking, the image was also resampled to 1 × 1 pixels (1 × 1 cm), such that areal measurements of classified sections could be obtained by counting the number of pixels, as long as the section is part of the primary vertical plane from which the GCPs were measured. This resample results in a large increase in the number of pixels; however, in test cases, this had little to no noticeable effect on the actual distribution of temperatures within the image. The mean, median, maximum, and minimum temperatures remain unchanged.

The result of this process is a paired set of visible and thermal images for each facet being considered, with the exception of traverses 7 and 8, at which point there are no shadows to consider, and the visible imagery becomes unnecessary.

#### Classification

The resultant thermal/visible image pairs were manually classified in ArcGIS. To classify each image, polygons were drawn on homogenous surfaces on the visible image, and an attribute table was populated. Each table contained nine nominal variables: traverse number, house number, orientation, shaded, shade source, covered, type, material, and colour. Traverse number is an integer 1 to 8 and references the traverse number from Table 3. House number is an integer 1 to 182 and references the house number in the study site. Orientation is a character N, E, S, or W and represents the direction the facet faces. Shaded is a binary number 0 or 1 and determines whether the pixels in the polygon are sunlit (0) or shaded (1). Shade source is a character string that states the source of shade on that surface. In the dataset, only three shade sources are used: self (shading cast on the facet by its own geometry), tree (shading cast by a tree or other vegetation), and N/A (used when the surface is sunlit). When the solar azimuth is ± 90° of the facet orientation angle such that none of the facet is sunlit (e.g. traverses 1–3 for west-facing facets), all polygons are classified with the shade source ‘self’. The same is true for all night-time traverses. Covered is a binary number 0 or 1 that indicates whether the surface is underneath a porch or awning. Type is a character string that states the kind of surface. Examples of type are wall, window, door, roof, etc. Material is a character string containing a visually determined first-order descriptor of the surface material, such as brick, glass, shingle, vinyl, etc. This variable was selected to allow emissivity corrections on a per-material basis. Colour is a character string containing a descriptor of the colour of the surface and was collected so that, if desired, a first-order estimate of albedo could be assessed. These variables were selected to provide a large range of opportunities to subset and analyse the data.

Each thermal image was assessed for all unique combinations of the above-mentioned variables, and the resulting classified homogenous polygons were complemented by a series of residual polygons. Because there are significant portions of each facet that contain pixels that are not neatly classified or that are impractical to classify, these polygons do not cover the entirety of a facet. Separate, large polygons were drawn that encompassed the entire facet and given type, e.g. resid_wall, resid_roof, etc. This residual polygon layer was joined to the primary classification layer and thereby ‘filled in’ all the gaps between the previously classified surfaces.

**Fig. 4:**
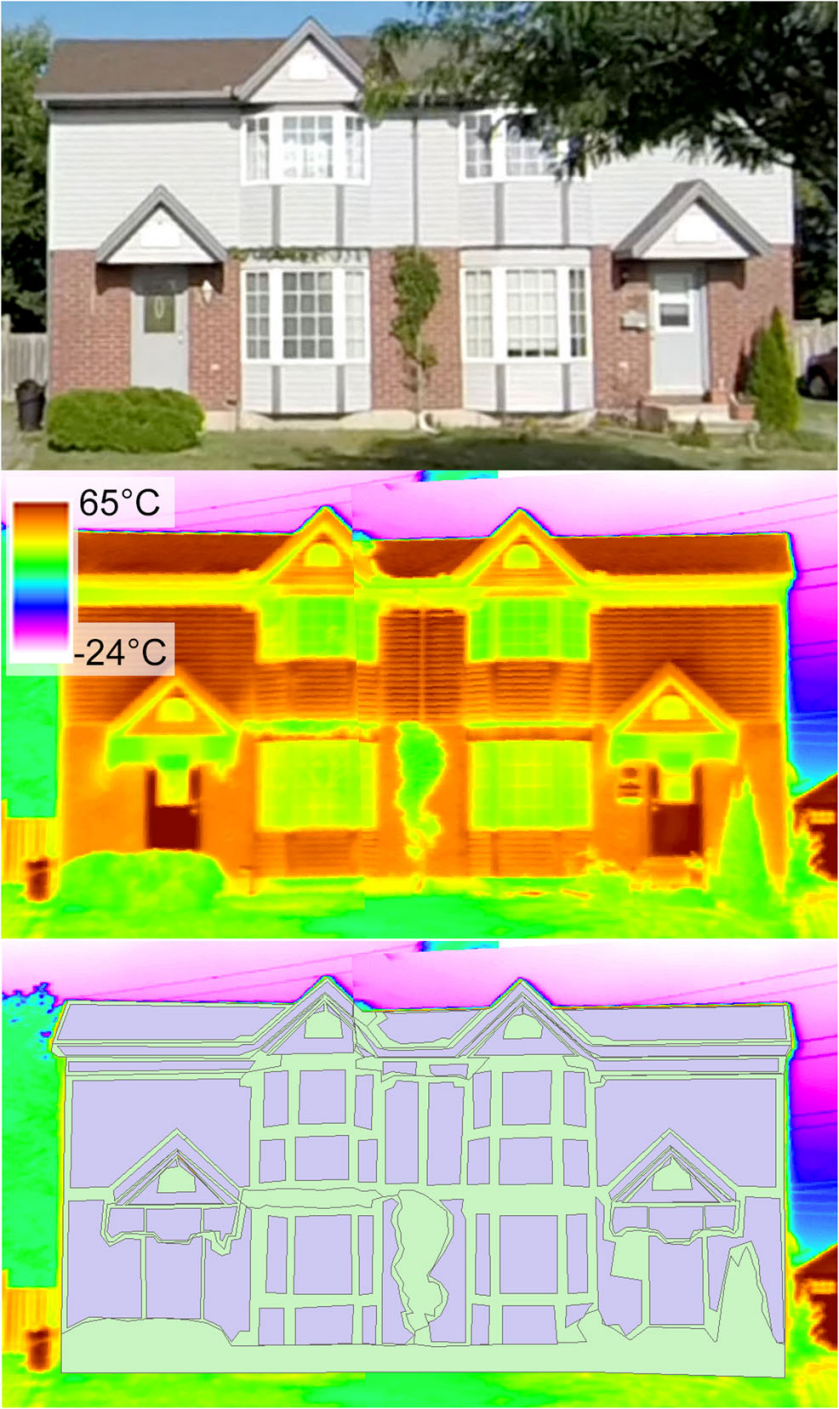
Visible image (top), thermal image (middle), and classified image (bottom) of house 169 (west-facing) during traverse 5 (approximately 16:50 EDT). Purple polygons are non-residual and green polygons are residuals, shown here before merging for illustrative purposes. There are 62 non-residual polygons with 9 unique variable combinations and 10 residual polygons with 5 unique variable combinations

Figure 4 shows an example of a thermal image used for classification as well as the associated drawn polygons. Clear shading patterns may be seen beneath the second storey eavestroughs and cast on the first storey wall by the awnings over the doors. Windows appear cool due to their lower emissivities as well as their transmission of solar radiation. Some obstructing vegetation can also be seen, notably in the middle of the first storey as well as to both lower corners of the house. The seam of the overlapped thermal images runs through the left windows and causes some distortion in the upper left triangular awning. Differences in wall materials can also clearly be seen. The second storey of this house is vinyl clad, and this material causes a clear horizontal striping of temperatures. The temperature distribution of this material has a greater range than the brick surface below it on the first floor. The lower portion of Fig. 4 shows the non-residual (purple) and residual (pale green) polygons for this thermal image. Residual polygons represent surfaces that are sufficiently small or too complex to classify or too near the boundaries of the structure. Glass surfaces do not provide useful radiometric temperature values and are excluded from consideration of surface temperatures in this paper.

The thermal image was then clipped to the extent of the polygons and converted to points whose value was equal to their temperature. The point file was spatially joined to the polygons such that each temperature point was then associated with the attribute table of the polygon in which it was located. This table of temperatures and nominal variables, referred to in this paper as an output table, was then exported as a CSV.

After additional formatting corrections, the output tables were joined in a final master dataset containing approximately 400,000,000 rows and 11,000 unique variable combinations. There remain some errors in the dataset that are the inevitable result of error when manually classifying such a large dataset. Tests for some types of errors, such as variable combinations that are logically inconsistent, yield combined errors of less than 2% of all points. It is not possible to quantify the magnitude of all the possible labelling errors, but based on the results of the tests performed, it is assumed to be small.

## Analysis and results

### Facet scale

#### Sample house and temperature distributions

**Fig. 5:**
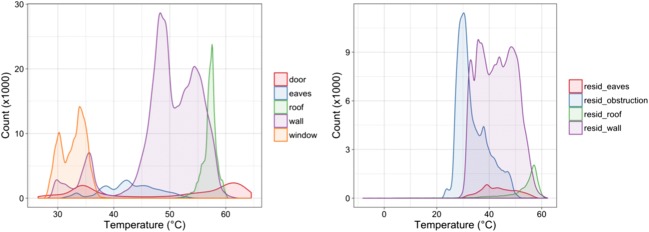
Smoothed temperature distributions coloured by ‘Type’ for the non-residual (left) and residual (right) classes from house 169 and traverse 5 (16:50 EDT). Note different x- and y-axes

Figure 5 shows smoothed temperature distributions output by the polygons from Fig. 4 coloured by their ‘Type’ variable. The non-residual types used are wall, door, roof, eaves, and window (included for illustrative purposes) and the residual types are residuals of wall, roof, eaves, and obstruction. The roof class is the only unimodal distribution, as this surface is uniformly sunlit. The door class has two distinct modes, corresponding to the sunlit body of the door that occupies the highest temperatures, as well as the shaded body of the door and the inset pane of glass, which comprise the second mode just below 35 °C. There are four distinct modes to the wall class which correspond to the sunlit and shaded components of the brick and vinyl siding. The highest mode at approximately 55 °C is the sunlit second storey. The second mode with the highest count is the sunlit brick of the first storey. The third mode at 35 °C is the shaded vinyl, and the final mode at 30 °C is the shaded brick. Separating the plot by the Material class would show this more distinctly, along with the wider range of the vinyl distribution compared to the brick. The window class also shows two distinct modes, which likely correspond in part to the panes of glass themselves compared to the frame in which they are set.

The residual classes show similar ranges of temperature compared to their non-residual counterparts. The residual roof class extends to lower temperatures than its non-residual pair, and indeed it is common for a few sky pixels to be caught in this class. For this reason, the non-residual polygons are never drawn right to the edge of a surface. The residual obstruction class trends cooler than the others as this class contains vegetation that obscures part of the facet and which remains cooler than most built surfaces.

The complex multimodality of the residual wall class demonstrates why surfaces are separated into non-residual and residual classes. It is possible to combine both into a complete histogram of radiometric temperatures of the facet, but it is also possible to separate out clear patterns in constituent materials and surfaces that allow for a clearer understanding of how each facet is behaving at the sub-facet scale.

#### Self-shading

**Fig. 6:**
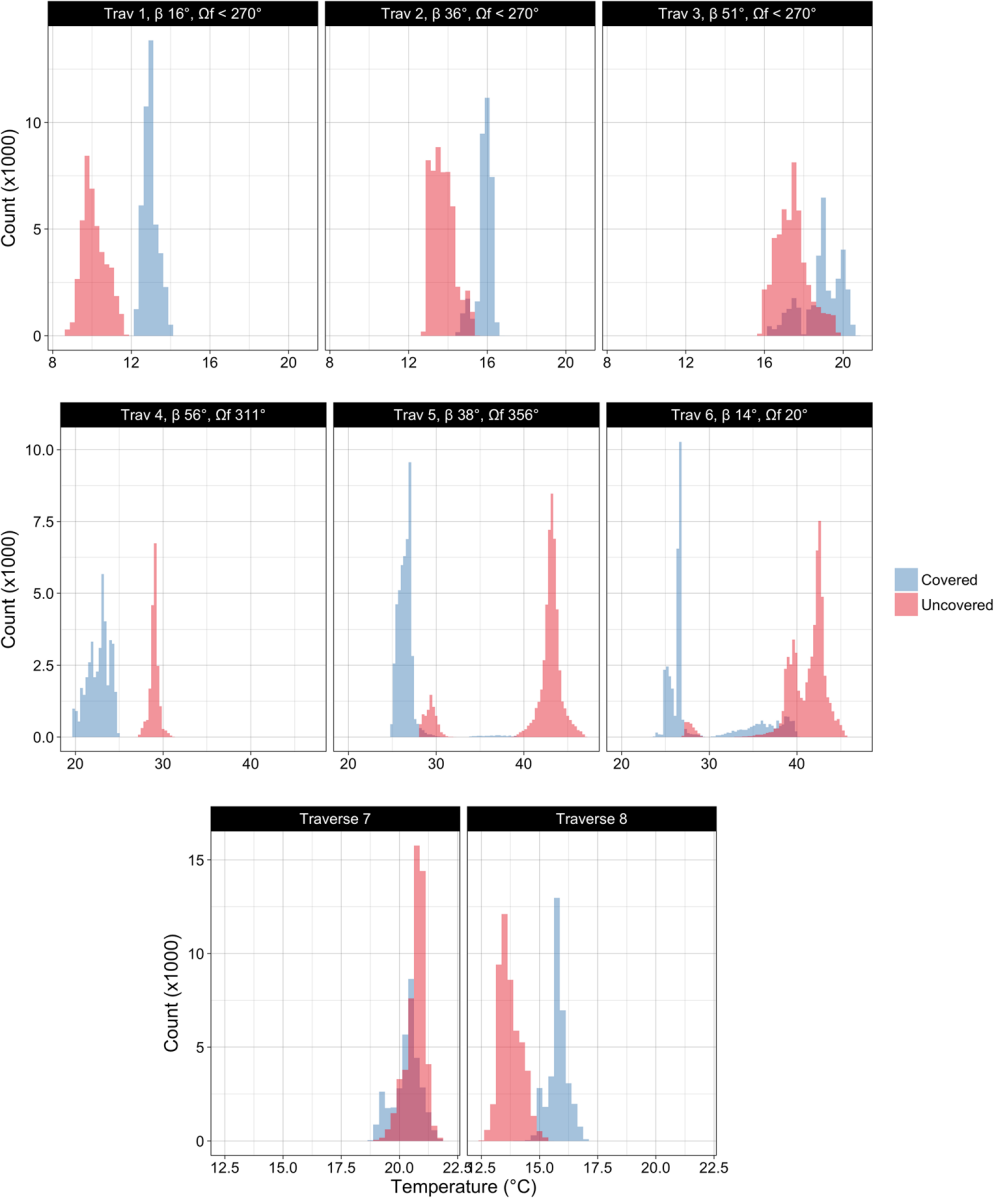
Histograms of all traverses for house 29 showing differences in covered and uncovered temperature distributions. β is solar elevation and Ω_f_ is solar azimuth relative to the facet (Ω_f_ < 270 means the facet is not sunlit). Bin width is 0.25 °C, note differing *x* and *y* scales for each row

At the facet scale, it is clear that sub-facet geometries that cast self-shade serve to reduce the surface temperature of the shaded surface and therefore the mean temperature of the entire facet. In S1 the large first-storey porches have additional effects on the facet temperature distributions. Figure 6 shows facet temperature distributions for a representative west-facing house in S1 for polygon type ‘wall’ and coloured by the ‘covered’ variable: covered surfaces are those underneath the first-storey porch. In the morning traverses (1–3), the entire facet is shaded, and surfaces covered by the porch are warmer than the uncovered surfaces. The distributions begin to overlap during traverse 3 as ambient air temperatures also climb. When the house receives direct solar radiation during traverses 4–6, covered surfaces are significantly cooler due to the shading effect provided by the porch. During the overnight traverses the distributions overlap and switch position. The reduction in SVF of surfaces underneath the porches reduces the rate and extent to which they cool, moderating temperatures under the porch, while surfaces above the porch experience much larger diurnal temperature ranges. Similar effects are seen at the facet scale for each orientation, though with differing timing dictated by sun-surface geometry.

One contributor to the high uncovered daytime temperatures is a secondary heating effect from the porch. The geometry of the porch roof reflects additional short-wave radiation to the wall surface above it, and its high surface temperature increases incident long-wave radiation on the above-porch wall surface. Comparing S1 wall temperatures of surfaces of similar height and material on houses with porches compared to houses without porches suggests that the porch may increase the wall temperature above it by as much as 6–8 °C when directly irradiated. The daytime warming effect caused by porch overhangs suggests that when comparing a neighbourhood with porches to one without porches, we should not only expect reduced mean temperatures from the shading but a greater range of temperatures across the facet and higher maximum wall surface temperatures due to the augmented short and long-wave loading from the porch roof. This secondary heating effect is much smaller than the impact of shading. Shading affects a larger area and yields a larger temperature change.

The degree of self-shading during each traverse is smaller in S2 than S1 as expected but nevertheless has a significant effect on mean facet temperatures. The geometries of S2 only appear to have reduced surface temperatures, and no secondary heating was noticed.

### Canyon scale

#### Self-shading fraction

**Fig. 7:**
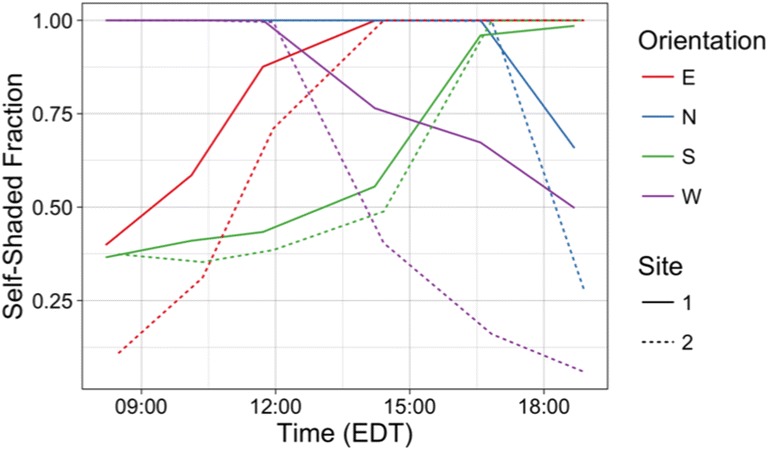
Self-shaded fraction for each street canyon

For each canyon orientation in both study sites, the number of self-shaded pixels was divided by the total number of pixels (less the roof classes) to provide a self-shaded fraction (i.e. a self-shaded fraction of 1.0 indicates a completely self-shaded facet, 0.0 indicates entirely sunlit) (Fig. 7).

The differences in site geometry can be clearly seen as S1 consistently has a greater degree of self-shading than S2. These differences are most apparent in west-facing facets, though the effects are clear in each orientation. The smaller difference in south-facing facets between the two sites is likely indicative of direct short-wave radiation reaching surfaces under porches (especially in the early morning) due to solar geometry, as well as the smaller number of porches on Dufferin Ave. compared to Woodman Ave. The effects of the rotated street grid are also clear in the asymmetry of the north and south facets. In a true north-south street grid, the north-facing facets would be irradiated briefly in the morning and late afternoon and the south-facing facets would see sinusoidal symmetry in their diurnal cycle. It is important to note here that because not every house was kept for analysis (see Section 2.3.1), these fractions are not accurate for the entire canyon per se, though they are accurate for the temperature measurements that are used for analysis.

#### Self-shading temperature reduction

**Fig. 8:**
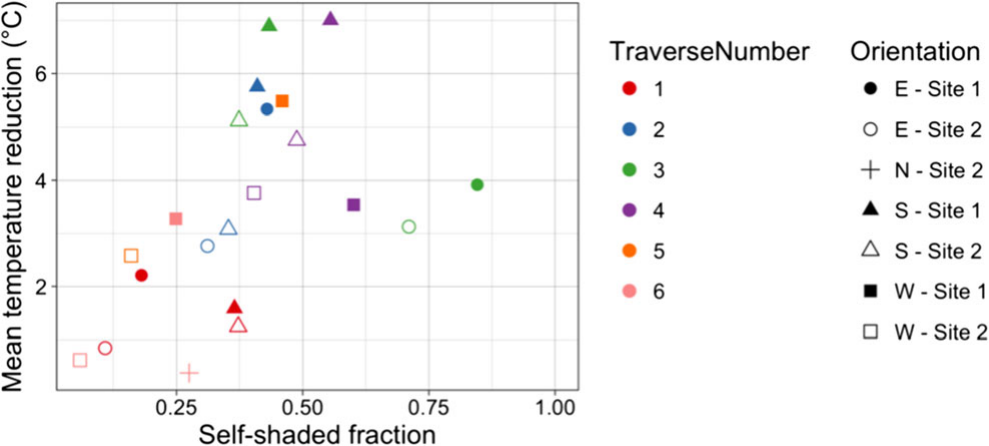
Self-shaded fraction vs. mean temperature reduction for each facet orientation and traverse number

If we assume that a facet with no sub-facet scale geometry will have the same mean facet temperature as the sunlit portions of the observed facets, we can estimate the reduction in mean facet temperature caused by self-shading by considering the difference in means of the sunlit portion and the sunlit portion combined with the self-shaded portion. Aggregating this concept of reduction in mean facet temperature to the canyon scale produces a relationship between self-shaded fraction and temperature reduction as shown in Fig. 8. Fitting a simple linear model to the relationship is significant at α < 0.05, with an R^2^ value of 0.35. A stronger relationship does not emerge if we decompose this to separate facet orientations, and it is probable that other sub-facet scale features such as material variability are playing a role. The outlying points for east-facing facets during traverse 3 may be due to the canyon-solar geometry during this traverse. The solar azimuth angle increases from roughly 140° to 145° during this traverse, resulting in a facet-normal azimuth of 70° to 75°. This may result in rapidly increasing self-shaded fractions for the facets, while the thermal response lags. Temperature reduction in S2 tends to be less than S1, even for similar degrees of self-shading. This is perhaps due to persistent thermal effects of surfaces that are consistently covered under the large porches of S1 and suggests that the duration for which a surface is shaded is also important in determining the mean temperature reduction. Thermal persistence effects related to shading history are also noted by Meier et al. (2010).

### Neighbourhood scale

**Fig. 9:**
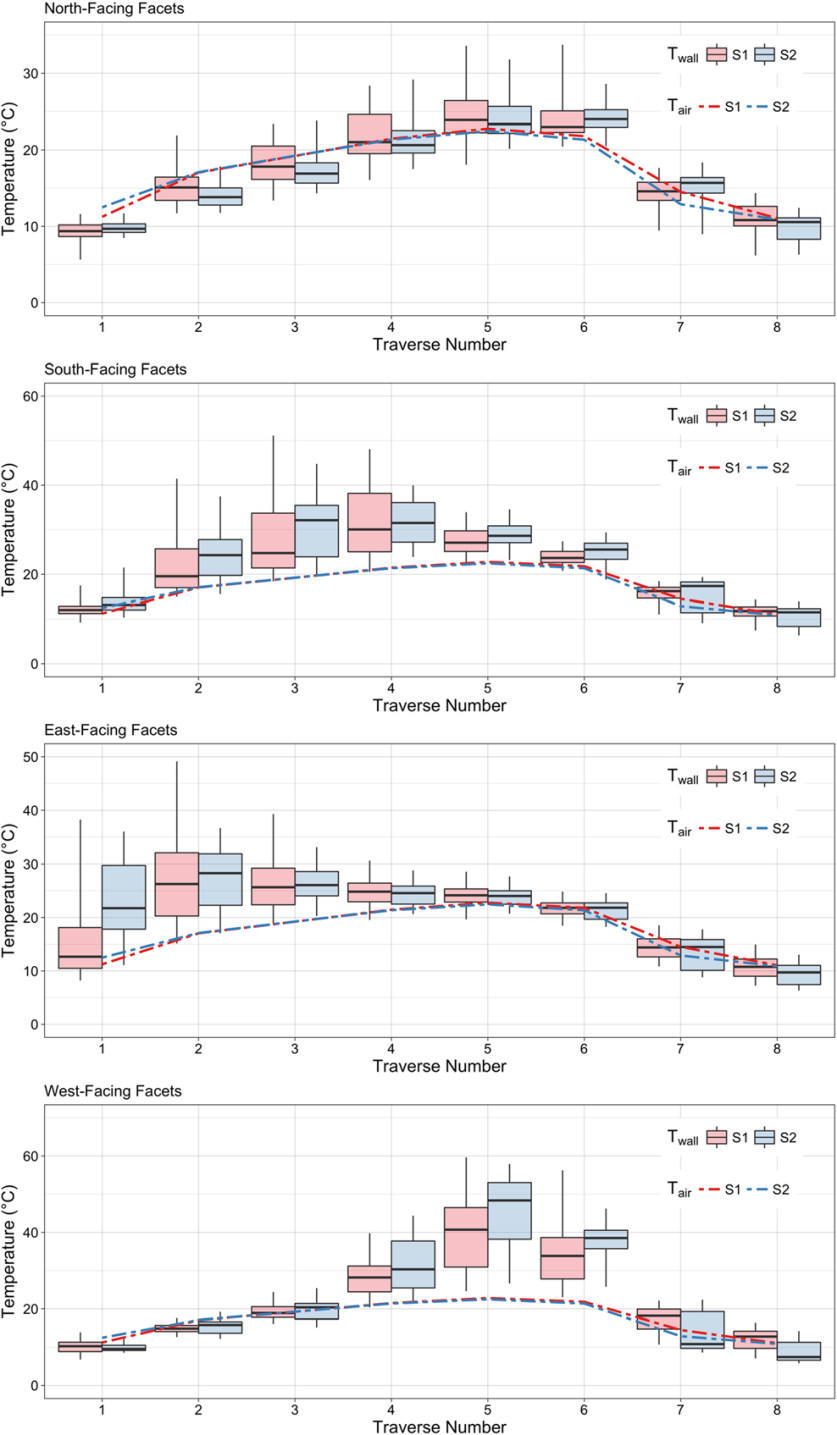
Comparison of surface brightness temperatures between sites 1 and 2 (boxplots) overlaid with mean air temperature at each site (line). Boxplot whiskers show the 98th and 2nd percentiles, the extent of the box shows the interquartile range (IQR), and the thick line in the box shows the mean. Note differing *y*-axes for each plot, and that traverses are *not* evenly spaced through the day as suggested by the *x*-axis; refer to Table 3 for precise timings

Figure 9 shows surface brightness temperature distributions of vertical facets for both neighbourhoods along with the canyon air temperatures.

There is some difference in air temperatures between the two sites. Air temperatures between S1 and S2 differ by < 0.5 °C with the exception of traverse 1 when air temperature at S2 is ≈ 1.25 °C higher than S1 and traverse 7 when S1 is ≈ 1.5 °C warmer than S2. The former is largely explained by a rapid morning warming of air temperature that corresponds with that observed at a local meteorological station and the latter suggests a slower rate of cooling in S1 compared to S2. The similarity of the air temperatures suggests that the differences seen in wall temperature distributions between the sites stem from geometries and materials of houses themselves rather than from neighbourhood-scale morphological characteristics. Small daytime differences in air temperature between the sites are also more broadly characteristic of studies that have examined air temperature differences between LCZs (Fenner et al. 2017; Beck et al. 2018; Kwok et al. 2019), likely due to enhanced turbulent processes that provide better mixing of the canopy-layer air by day.

The inter-neighbourhood comparison of north-facing facets is quite interesting as it shows the effect of porches mostly in the absence of direct sunlight. Some short-wave radiation will be received via reflection from the opposing canyon walls as well as a diffuse sky component, but they are only directly irradiated during traverse 6. These facets generally show a higher mean temperature throughout the day for S1 compared to S2, as well as a larger interquartile range but similar range. This suggests that in the absence of direct insolation, the porches may serve to trap heat. When the surfaces are finally sunlit, S2 achieves a greater mean temperature, likely due to the greater amount of self-shading in S1. During the night, the increased mean temperature in S2 carries forward through traverse 7 before the mean temperatures roughly level out by traverse 8, although the IQRs suggest that temperatures skew higher in S1. The hypothesis that above-porch temperatures are increased by reflected short-wave and emitted long-wave radiation from the porch to the wall surface above is likely not the source of the higher temperatures in S1 from traverses 1 through 5 as there is no direct sunlight to reflect. Instead, this difference is perhaps due to a persistent effect of decreased SVF in S1, which is supported by the slightly higher air temperatures and slower rate of air temperature cooling.

The south-facing facets show decreased daytime mean temperatures with a greater IQR for S1 consistent with more facet surface structure, though larger range, and lower mean temperatures during traverse 7 that level out by traverse 8, suggesting a slightly slower rate of cooling for S1. This slower cooling rate is likely an effect of the smaller SVF for covered walls in S1. The IQR of S1 when shaded (traverses 6–8) is somewhat smaller than for other orientations, which may be related to a greater number of single storey houses on the north side of the street which result in more homogenous covered/uncovered temperatures.

The east-facing facets show a sharp increase in temperature between traverses 1 and 2 when incident solar radiation is most normal to the facet. As the solar zenith angle decreases and the angle between the sun and facet increases, so too does the amount of shaded area. Received short-wave radiation decreases enough that there is a slight cooling of mean temperatures between traverses 2 and 3, as well as a reduction in the range and IQR. All three sunlit traverses show a decreased mean temperature in S1 compared to S2. Once the facets become shaded, however, the means, ranges, and IQRs are very consistent between the sites during the remaining sun-up traverses (4–6). Traverse 7 shows similar mean temperatures with a smaller IQR for S1, and by traverse 8 the mean temperature for S1 is slightly higher than S2.

West-facing facets show a very small temperature range through the morning when shaded, and during the sunlit traverses 4–6 echo, the patterns seen in the other orientations. Interestingly the night-time temperatures for S2 skew quite cool, and as such the mean temperatures for S1 are consistently and significantly warmer through the night.

It is important to note that each orientation in Fig. 9 is displayed on different *y*-axes. The warmest temperatures by far are found on the west-facing facets, reaching 60 °C in S1, where night-time and shaded temperatures hover near 10 °C. South-facing facets experience a similar amplitude of temperatures by virtue of being sunlit through most of the day. East-facing temperatures are slightly less extreme in their range, which is likely due to the reduced total insolation they receive as a result of the rotated street grid. Most muted is the cycle of north-facing facets, ranging from means of 10 °C to 25 °C, roughly equivalent to the ambient air temperature by virtue of not experiencing significant direct solar radiation.

### Opposing wall temperature differences

**Fig. 10:**
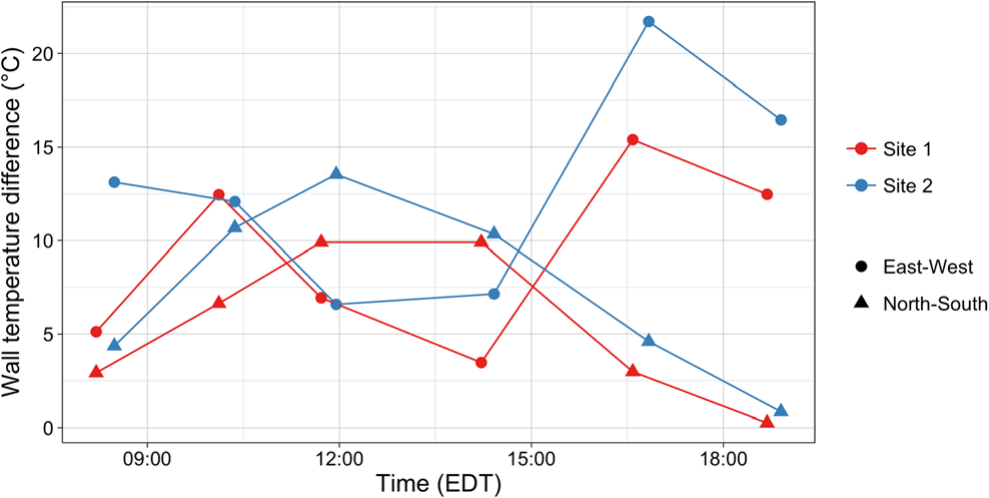
Magnitude of opposing wall temperature differences for each site and canyon. Note that canyons here are named by facet orientation rather than street direction, i.e. east-west refers to the difference between east- and west-facing facets rather than the street that runs east to west

Krayenhoff and Voogt (2016) suggest that temperature differences between opposing canyon walls are an important forcing of effective thermal anisotropy as observed by airborne or satelliteborne sensors. Opposing wall temperature differences were quantified for each site and canyon orientation. The magnitude is determined by taking the absolute difference of mean temperatures between opposing canyon walls. Figure 10 displays these results. Sun-up traverses are considered, and observations are subset to remove roof, window, and residual classes.

Temperature differences between opposing walls range from less than 1 °C to over 22 °C. With the exception of traverse 2, the magnitude is greater for S2 than for S1. The large area in S1 that is covered by porches moderates the temperatures between canyon walls and keeps the mean temperatures of opposing walls more similar than S2. During traverses 1 and 2, both sites have greater wall temperature differences in the E-W canyon than the N-S canyon. This pattern switches for traverses 3 and 4, when the magnitude of the N-S temperature difference is greater than E-W for both sites. During traverse 3, the east-facing facets in both sites become > 50% shaded, and during traverse 4, the west-facing facets begin to receive direct solar radiation. These contribute to a smaller solar forcing of temperature differences and reduce the magnitude of the temperature difference. In traverses 5 and 6, the E-W canyons exhibit the highest difference as the west-facing facets become completely irradiated.

The N-S facets show a pattern of increasing temperature differences from the morning through to solar noon at which point differences decrease. The E-W facets show the inverse pattern, starting higher, decreasing to solar noon, and then increasing, though the first traverse in S1 clearly does not follow this pattern. Additionally, traverse 4 in S1 shows a decreased magnitude from traverse 3 while S2 shows an increase. The difference in the procession of the temperature difference is likely due to the different sub-facet geometries between the two sites, which keeps the mean temperature of irradiated S1 walls lower than in S2.

The opposing wall temperature difference is higher among N-S facets in S2 than S1 at all times of the day. The E-W facets also have a higher difference in S2 with the exception of traverses 2 and 3, during which the magnitudes are very similar (< 1 °C difference) and slightly higher in S1.

## Discussion and conclusions

### Urban climate studies

The diurnal procession of mean temperatures by orientation follows those described by Voogt and Oke (1998) with some modification due to street grid rotation. This research also highlights the complexity of sub-facet temperature distributions and the strong reliance of temperature on both individual material components as well as solar geometry as demonstrated by Aldred (2003).

The facets examined in this paper may be considered relatively complex in their sub-facet distribution of materials in that within each canyon there are many different materials as well as windows, doors, etc. and no obvious repeating pattern that may be found in a downtown street canyon like those referenced in much of the introduction. These variations in material and location impact surface temperatures, and at an instant of time and over a diurnal cycle, these complexities are important in understanding sub-facet temperature distributions. Careful consideration of the sub-facet scale material and geometric composition is important to characterizing the vertical surface temperatures of a canyon. Sampling of some material(s) and not others could lead to biased results. The location of a sampling area in relation to the facet geometry and diurnal procession of shading patterns could similarly create bias. An appropriate degree of consideration must be tailored to the scale of the subject being investigated as well as the instruments performing the observations. Sampling of small portions of walls with FOV-averaging instruments does not easily allow sub-facet variability assessment nor can it determine the within-FOV variability.

### Tree shading vs. self-shading

**Fig. 11:**
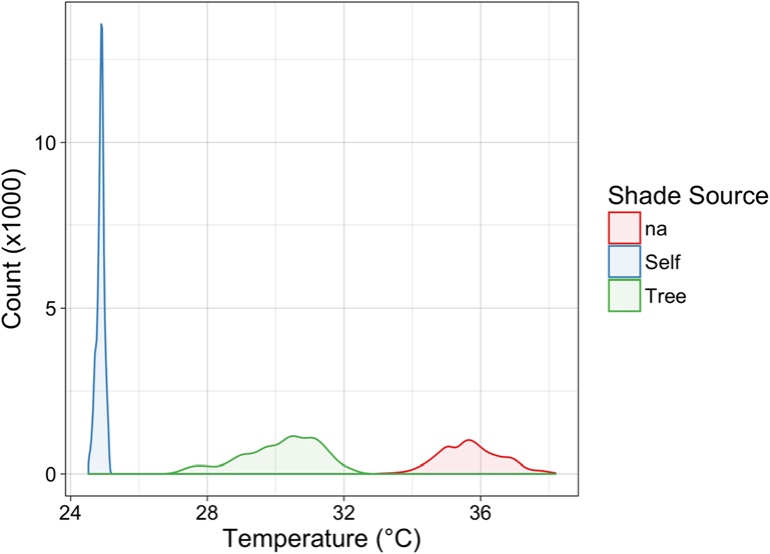
Temperature distributions of sunlit (shade source *na*), self-shaded, and tree-shaded wall surfaces on house number 31 from traverse 6. All temperatures are of the same red brick wall

This research specifically sought out neighbourhoods with a small number of in-canyon trees to reduce the impact of tree shading on wall facets. Nevertheless, S1 still contains a number of large trees whose height is greater than the houses. At certain solar angles, these trees shade large portions of the canyon and thereby reduce mean wall temperatures. There has been much attention paid recently to the implementation of trees and other vegetation to urban microclimate models (e.g. Nice et al. 2018b; Lee and Park 2008; Krayenhoff et al. 2014). While tree shading does lower surface temperature, the results of this research suggest that self-shading caused by geometric complexity may cause a greater reduction in surface temperature. This is not to say that the impact of tree shading is negligible or that self-shading is more important per se, but instead that when a given surface is self-shaded, its temperature is lower than when it is tree-shaded. Figure 11 demonstrates one such case where the differences in temperature distributions from different shade sources are clear.

To ensure that these differences are not influenced by surface thermal property variation, comparison is made only between identical surfaces (i.e. same type, material, colour, and covered variables) with differing shade sources. There are 34 such instances in site A. Figure 11 is an extreme illustrative example. Aggregated over all 34 instances, tree-shaded surfaces are on average 6.4 K cooler than unshaded surfaces, whereas self-shaded surfaces are on average 8.5 K cooler. Self-shaded surfaces also have a smaller median standard deviation (0.8 K) when compared to tree-shaded surfaces (1.7 K). In addition it is probable that there is a temporal trend similar to that explored in Section 3.1.2.

This is likely a consequence of many tree canopies only partial interception of direct short-wave radiation, whereas a constructed overhang intercepts 100% of direct solar radiation. Determining which shading source contributes more to an absolute cooling of facet temperatures at a canyon or neighbourhood scale depends on the relative degree of shading provided by each. Trees would need to shade a larger area than self-shading of the facet geometry in order to achieve the same temperature reduction. It has also been shown that porches heat the wall surface above them, which reduces their efficacy slightly at a facet scale. One advantage of the tree cooling is that it does not produce this secondary heating effect. Additionally, trees can shade pedestrian walkways and roads to lower other surface temperatures within the street canyon.

### Generalizing results

It is shown here that sub-facet scale structure decreases facet surface temperature when it casts shade on the facet and that certain structures increase surface temperature relative to a smooth facet. This paper examines the impacts of eaves and eavestroughs and porches as sub-facet obstructions.

Generalizing from our results, it is hypothesised that during the day, geometric obstructions extending from the wall surface will self-cool commensurate with their ability to cast shade. If the obstruction extends from the middle of the facet, e.g. a first-floor porch on a two-storey house, it will also induce a secondary heating effect by reflecting short- and emitting long-wave radiation. The magnitude of this heating effect will depend on the radiative and thermal properties of the reflecting surface. When there is no wall surface above the obstruction, this radiation is reflected and emitted back to the atmosphere. During the night, they will tend to increase wall temperature commensurate with their ability to lower the sky view factor of surfaces below them.

One of the most common larger-scale vertical obstructions that is likely in urban environments is the balcony. The results of this research suggest that on, e.g. an apartment building with many balconies, we would see a general reduction in mean facet temperature due to shading cast by these obstructions on the surface as well as a slight warming of the wall surface above the balcony. Indeed it is also possible that during low solar elevations, tall buildings may experience shading above the balcony itself and a slight warming of reflected radiation below it. A balcony with solid siding may cast additional shade on the surface of the balcony itself and reduce heating above the balcony surface as well as potentially reduce convective transfer of heat and modify air flow regimes.

Sub-facet geometry may not only consist of horizontal obstructions such as porches but of textured facades along the vertical axis, but to similar effects. It would be expected that the spatial distribution of shading and consequently temperature would then be controlled largely by solar azimuth rather than elevation.

The effect of self-shading will be greater in canyons with lower H:W ratios as these will tend to isolate the canyon walls from shading effects of the opposing canyon wall. Smaller H:W canyons will spend larger portions of the day with their walls completely irradiated, whereas the larger the H:W ratio, the longer the time that the canyon wall will be shaded by the opposing wall. Residential neighbourhoods with low density housing likely have the greatest influence of self-shading due to low canyon H:W ratios and potentially complex sub-facet geometries. High density LCZs where canyon walls may be shaded for much of the day by the opposing canyon wall will likely see diminished magnitudes of self-shading temperature reductions.

### Computational fluid dynamics

Differential heating of canyon walls has been shown to impact within-canyon circulations (e.g. Xie et al. 2005a, 2007; Cai 2012; Magnusson et al. 2014; Nazarian et al. 2018). Using Computational Fluid Dynamics (CFD), canyons of varying geometric properties are heated on one wall under various atmospheric stability conditions and above-canyon wind flows, and the influence on within-canyon air flow and pollutant dispersion is studied. Results suggest that heating of the leeward wall somewhat reduced the magnitude of the canyon vortex, while heating of the windward wall can produce secondary vortices within the canyon under certain H:W ratios. The Xie et al. studies define the degree of wall heating as ∆θ = T_wall_ − T_air_ and consider values of ∆θ of 0.2 to 15 K. Similarly, Cai (2012) examines values of ∆θ of up to 15 K and Magnusson et al. (2014) values of approximately 10 K. The walls are heated with a uniform temperature, and higher values of ∆θ have a greater degree of influence on within-canyon flow. Results from this research suggest that values of ∆θ much greater than 15 K could be considered, peaking at ≈ 27 K during traverse 5 in S2. Incorporation of sub-facet scale material variability could push portions of the wall facet well over a 27 K difference, though the lower H:W ratios of this study may promote greater values of ∆θ. The previous studies also leave the non-heated wall at air temperature. Results from this research suggest that during the day, shaded wall facets are generally slightly above within-canyon air temperature due to reflected short-wave and long-wave radiation received from the sunlit canyon walls.

Incorporation of sub-facet scale temperature variability may also impact within-canyon wind flows. Differences in opposing canyon wall temperatures of < 5 K are shown to impact within-canyon wind flows (Xie et al. 2005a, b), and this research has observed within-facet temperature differences of > 20 K. It is probable however that the presence of the structural complexity that causes these temperature differences would have a greater influence on wind flows within the canyon. The results of this research may be useful for setting realistic conditions for future CFD studies, especially if similar studies are performed in 3D. Mean wall temperatures are likely to be biased high if sub-facet influences are not taken into account and ∆θ may also bias warm.

### Implications for thermal anisotropy

Results from modelling studies (Hu and Wendel 2019; Wang et al. 2018; Krayenhoff and Voogt 2016) suggest that temperature differences between walls can be an important contributor to thermal anisotropy over urban surfaces. Further, the representation of these facets in models that estimate surface temperatures typically does not incorporate any sub-facet structure that would result in a decrease in temperature of sunlit facets or warming of shaded facets. Our results suggest that sub-facet scale geometries and material variability do contribute to temperature differences between opposing canyon walls as there are significant differences between the two sites in opposing facet temperatures that are not explained by canyon geometry alone. From a canyon scale geometry perspective, the two sites examined are relatively similar: simulating the differences in their canyon scale geometries alone does not account for the significant temperature differences between the sites. This suggests that sub-facet scale geometries may have an impact on effective thermal anisotropy as measured by airborne or satellite sensors. The linear increase of anisotropy to canyon geometry at small H:W ratios identified in Krayenhoff and Voogt (2016) may be modified if sub-facet structures provide substantial control on wall temperature decreases in more open canyon geometries.

Effective anisotropy would be increased the most when the greatest degree of facet self-shading is caused by the smallest amount of geometric complexity. At small solar zenith angles, a small roof overhang can cause large amounts of wall shading. A large porch at similar angles will cause more self-shading due to its greater size but also thereby receive a significantly larger amount of insolation. Shading will only reduce effective anisotropy insofar as the sensor is able to view this shading. Therefore, even though a large overhang will reduce mean wall temperature, it must be considered from a sensor view model to determine the impact on measurement of thermal anisotropy.

### Limitations

As discussed in Section 2.3.1, a large number of houses were eliminated from consideration due to failure of the KLS and facet obstruction. This limits the dataset’s ability to be an accurate representation of all temperatures in the neighbourhood. At solar azimuth angles in which a tree that obstructs our imager’s view of a facet also stands between the sun and the facet, the mean surface temperature of that facet should be much lower than the canyon average, due to the shading from the tree. This should not have an impact on self-shading temperatures but does affect the actual ‘complete’ canyon surface temperature of the neighbourhood and is not captured by the observations. As an example, during traverse 1, complete east-facing surface temperatures for S1 are likely lower than the dataset suggests, because a number of rejected facets will have trees casting shade on them. No field site will ever be perfect, but future uses of the method should strive to find different facet geometries with similar degrees of vegetation so that this is not a confounding variable when comparing sites. It would also simplify the comparability of the results to find a street grid with a true North-South orientation.

An additional limitation of the method is that determining the proportion of shaded area of the facet requires the inclusion of the residual classes. Because the non-residual polygons are not drawn to the full extent of the surface being classified, it is only possible to determine the degree to which an entire facet is self-shaded, and it is not possible to accurately assess the degree to which an individual material is self-shaded at the sub-facet scale. This is frustrating because it is hypothesised that the relationships between self-shaded facet fraction and temperature reduction (Fig. 8) will be stronger when subdivided by surface material to account for differing thermal properties. If the degree of self-shading could be quantified at the sub-facet material scale, and mean temperature reduction considered at this scale, a stronger relationship would be expected. This could be achieved by reclassifying the imagery with this goal in mind, though a complete reclassification would require significant work.

Perhaps one of the largest drawbacks of the method is the large time commitment necessary to manually process the data. Many processes could be reasonably automated or at least performed significantly faster with the aid of scripting; however, some primary tasks such as geo-referencing of each individual image must be performed by hand. The most time-consuming portion was the manual classification of thermal images. Once the method had been tuned and some proficiency attained, it took approximately 2.5 to 3.5 h per house depending on the complexity. For the pruned dataset of 125 houses, this amounts to roughly 310 to 435 h, or 40 to 55 eight-hour days comprised entirely of classifying. Recently the intersection of machine learning and massive datasets of hemispherical street-level imagery like Google Street View have been used to characterize canyon parameters like SVF and vegetated fraction (Gong et al. 2018; Middel et al. 2018) as well as predict mean radiant temperatures (Nice et al. 2018a). A similar process could likely be used to help characterize sub-facet scale material and structural variation.

### Future work

There are many potential avenues for future work that have been identified through this research. Firstly, because most urban canopy models lack representation of facet scale structure and thereby self-shading of their facets, a simple parameterization of the degree of self-shading and reduction in mean facet temperature could be implemented in such a model. Indeed, some microscale models (e.g. SOLWIEG, Lindberg et al. 2015) include such effects in their parameterization of surface temperature. Alternatively, implementation of sub-facet scale geometries could be considered: an eavestrough or porch could feasibly be modelled to some degree of accuracy while still falling within, e.g. TUF-3Ds requirement for plane-parallel cubic geometries. This would allow the impact of self-shading to be examined at a variety of neighbourhood geometries, sub-facet scale geometries, latitudes, times of year, and if coupled with a model such as VTUF, types of vegetation as well. Acquiring observations with a similar method in different LCZs as well as in neighbourhoods with different degrees of vegetative shading could help address some of the questions and hypotheses posed in the discussion. Opposing canyon wall temperature differences are just one component of effective urban thermal anisotropy. The full implications of sub-facet scale geometries would need to be considered in a sensor view model to truly tease out the implications of these complexities for thermal anisotropy.

Further topics that could be addressed using the assembled data are material-scale temperature variations, or differences between self- and tree-shading, which have only been speculated on here. Additional micro-meteorological variables such as road temperature were collected on the mobile platform but not included in this paper: these could be examined and coupled to the present research. If relationships exist between, e.g. road temperatures and wall temperatures, perhaps parameterizations of wall temperatures could be obtained for (near) nadir sensing geometries and lead to improved assessment of complete urban surface temperatures.

Finally, improvements to the method could be pursued. Classifying total shading at a material rather than facet scale would allow for clearer observation of the proposed relationship between sub-facet material characteristics, total shading fraction, and mean facet temperature reduction. Systems that couple thermal and RGB imagery with a LiDAR scanner such as Borrmann et al. (2012) or point cloud matching methods like Lin et al. (2019) present opportunities to collect synchronized data in an efficient manner while creating a dense 3D dataset that would be ideal for applications such as this. There also exists the possibility of using supervised classification methods on a well co-registered dataset to improve the speed at which classification could be performed, as this was identified as a significant bottleneck in the previous section.

### Summary

This paper presents a novel method of observing urban wall temperatures using an instrumented mobile platform. The method allows high spatial resolution datasets of urban surface temperatures to be obtained at a neighbourhood scale and allows examination of temperature distributions at a sub-facet scale. Two neighbourhoods in London, Canada, with differing sub-facet geometric complexity were observed to investigate the effect that such geometries have on vertical facet temperature distributions. The results suggest that at the facet scale:

1. Shading cast on a facet by its own geometric complexity reduces mean facet temperature.
2. Geometric complexities below building height may increase the temperature of the surface above them by day.
3. Large obstructions such as porches may alternately reduce mean facet temperature by day but increase it by night.

Observations made at the (sub-) facet scale were then scaled up and considered at the urban canyon scale. The results of this analysis suggest that:

1. Facet-scale patterns of decreased daytime temperatures due to shading and increased night-time temperatures due to large overhangs aggregate to the canyon scale.
2. Reduction of daytime temperatures correlates with the self-shaded fraction of the facet but is partially controlled by other factors.

Finally, the observations were once more scaled up to the neighbourhood scale, and the two sites were compared. The results of this analysis suggest that:

1. A neighbourhood with relatively larger obstructions at the facet scale will have lower daytime mean wall temperatures and a larger temperature range.
2. Greater sub-facet geometry tends to decrease opposing canyon wall temperature differences.
3. Neighbourhoods with comparable canyon scale geometries and characteristics (i.e. similar LCZs) may exhibit significantly different sub-facet temperatures due to their microscale geometry.

These results demonstrate the need to carefully consider sub-facet scale material and geometric complexities when performing surface temperature observations at these scales. Implications for modelling of in-canyon processes such as surface temperatures and wind flows were discussed, as well as possible consequences for measurement of effective urban thermal anisotropy. The research identified additional questions such as the relative importance of vegetative shading vs. geometric self-shading and presented suggestions for future work that could aid in better understanding the causes and magnitudes of the effect. The dataset created will remain a useful tool for future investigations in to sub-facet scale temperatures that can be used for model verification, analysed for additional insight, or even returned to and reclassified if desired, to improve some identified shortcomings.

## Figures and Tables

**Fig. 12:**
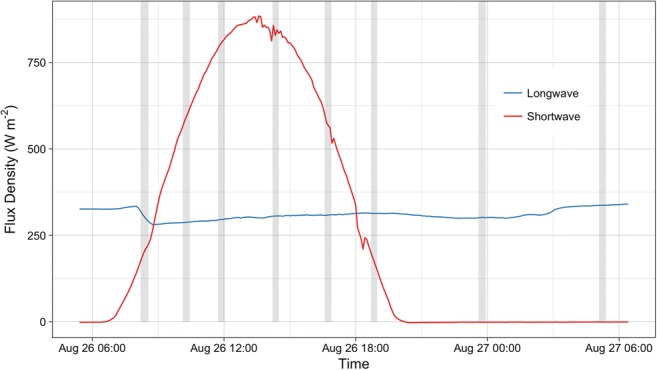
Short- and long-wave flux densities measured from the monitoring site (Section 2.2.1) during the observation period. The grey bars show time and duration of traverses

## Appendix

### Sky conditions

As previously noted, a day with clear sky conditions was deliberately selected to ensure that cloud shading would not impact facet temperatures. Figure 12 shows the incident short- and long-wave flux densities measured at the fixed monitoring site for the study period. Some scattered cloud effects are noticeable just after solar noon and again later in the afternoon in the short-wave signal. These cloud effects overlap with traverse 4, though no evidence of cloud shading was seen in the thermal or visual imagery from the field sites. The slight increase in incident long-wave radiation that occurs at approximately 0300 EDT on August 27 suggests the presence of some clouds, but any effect on cooling rates caused by this are assumed to be equal between sites 1 and 2.

### Influence of canyon H:W rati+o on surface temperatures

As noted in Section 2.1, the sites have slightly different average canyon H:W ratios. These differing H:W ratios will have some effect on facet temperatures, and TUF-3D was employed to estimate the magnitude of this effect. Two simulations were run with identical boundary conditions and surface radiative and thermal properties; one with H:W of 0.25 and the second with H:W of 0.15. Forcings were those obtained from the fixed monitoring site described earlier. The simulation was run for 48 h with the last 24 h considered for this comparison. Results are summarized below in Table 5.

**Table 5:** Summary of temperature differences caused by canyon H:W difference between S1 and S2

Facet	Temperature	difference	(°C)
	Maximum	Mean	Median
North	1.23	0.46	0.31
South	1.31	0.52	0.37
East	1.14	0.41	0.28
West	1.12	0.40	0.24
